# IL-4i1 Regulation of Immune Protection During *Mycobacterium tuberculosis* Infection

**DOI:** 10.1093/infdis/jiab558

**Published:** 2021-11-05

**Authors:** Lerato Hlaka, Mumin Ozturk, Julius E Chia, Shelby-Sara Jones, Shandre Pillay, Sibongiseni K L Poswayo, Thabo Mpotje, Justin K Nono, Simphiwe R N Simelane, Suraj P Parihar, Sugata Roy, Harukazu Suzuki, Frank Brombacher, Reto Guler

**Affiliations:** 1 International Centre for Genetic Engineering and Biotechnology, Cape Town Component, Cape Town, South Africa; 2 Institute of Infectious Diseases and Molecular Medicine (IDM), Division of Immunology and South African Medical Research Council (SAMRC) Immunology of Infectious Diseases, Department of Pathology, Faculty of Health Sciences, University of Cape Town, Cape Town 7925, South Africa; 3 Wellcome Centre for Infectious Diseases Research in Africa, Institute of Infectious Disease and Molecular Medicine, Faculty of Health Sciences, University of Cape Town, Cape Town, South Africa; 4 The Jackson Laboratory for Genomic Medicine, Farmington, Connecticut, USA; 5 Laboratory of ImmunoBiology and Helminth Infections, Institute of Medical Research and Medicinal Plant Studies, Ministry of Scientific Research and Innovation, Yaoundé, Cameroon; 6 RIKEN Center for Integrative Medical Sciences, Yokohama, Japan

**Keywords:** IL-4i1, *Mycobacterium tuberculosis*, immunity, host-directed therapy

## Abstract

**Background:**

Interleukin 4 (IL-4i1)–induced gene 1 encodes L-phenylalanine oxidase that catabolizes phenylalanine into phenylpyruvate. IL-4i1 is mainly expressed by antigen-presenting cells (APCs), inhibits T-cell proliferation, regulates B-cell activation, modulates T cell responses, and drives macrophage polarization, but its role in bacterial infections is understudied.

**Methods:**

We evaluated IL-4i1 deletion in macrophages and mice on infection with virulent H37Rv and W-Beijing lineage hypervirulent HN878 *Mycobacterium tuberculosis* (*Mtb*) strains. The bacterial growth and proinflammatory responses were measured in vitro and in vivo. Histopathological analysis, lung immune cell recruitment, and macrophage activation were assessed at the early and chronic stages of *Mtb* infection.

**Results:**

IL-4i1–deficient (IL-4i1−/−) mice displayed increased protection against acute H37Rv, HN878 and chronic HN878 Mt infections, with reduced lung bacterial burdens and altered APC responses compared with wild-type mice. Moreover, “M1-like” interstitial macrophage numbers, and nitrite and Interferon-γ production were significantly increased in IL-4i1−/− mice compared with wild-type mice during acute *Mtb* HN878 infection.

**Conclusions:**

Together, these data suggest that IL-4i1 regulates APC-mediated inflammatory responses during acute and chronic *Mtb* infection. Hence, IL-4i1 targeting has potential as an immunomodulatory target for host-directed therapy.

Alveolar macrophages are the first cells to phagocytose *Mycobacterium tuberculosis* (*Mtb*) on infection [1]. It has been suggested and demonstrated that *Mtb* can subvert the host protection by skewing macrophages to a less hostile, alternatively activated state to avoid classic effector killing functions [1–4]. Identifying target genes through transcriptomics studies can provide insights into complex host-pathogen interplay. Our group previously investigated the subverting strategies of *Mtb* by conducting a genome-wide gene expression analysis, using CAGE (Cap Analysis of Gene Expression) transcriptomics, on classically M1 (interferon [IFN] γ) and alternatively activated M2 (interleukin 4 [IL-4]/interleukin 13 [IL-13]) macrophages after *Mtb* infection [5–8].

Interleukin 4il (IL-4i1) is an L-phenylalanine oxidase that plays peculiar immunomodulatory roles in various types of tumors and inflammatory diseases. Its name is attributed to the early discovery of its expression in IL-4–stimulated B cells and has been implicated in the activation of B cells and T cells [9–12]. Subsequently, IL-4i1 was discovered to be a secreted glycosylated protein, expressed by antigen-presenting cells (APCs) including macrophages and dendritic cells [13]. The IL-4i1 enzyme mainly catabolizes phenylalanine into phenylpyruvate and produces toxic derivatives, including hydrogen peroxide (H_2_O_2_) and ammonia [13]. Products of IL-4i1 enzyme contain antibacterial properties which are mainly attributed to H_2_O_2_ and further amplified by the accumulation of ammonia [14, 15]. In addition, the IL-4i1–mediated catabolite production of H_2_O_2_ has been reported to down-regulate T-cell receptor zeta expression, resulting in the inhibition of T-lymphocyte proliferation [13]. More recently, it was discovered that secreted IL-4i1 can directly bind to human T lymphocytes, thus inhibiting effector T-cell proliferation, which is potentially independent of its enzymatic ammonia, H_2_O_2_, and phenylpyruvate activity [16].

Other studies reported the role of IL-4i1 in regulating macrophage polarization [17]. Amino acid catabolism with the production of toxic compounds can be a survival strategy used by the host against invading pathogens. Brain tissues from patients with tuberculosis meningitis and human immunodeficiency virus coinfection showed 25-fold up-regulation of IL-4i1 expression compared with controls [18]. Aerogenic infection with H37Rv *Mtb* resulted in the up-regulation of lung IL-4i1 messenger RNA (mRNA) expression in various mouse strains (C57BL/6, BALB/c, DBA/2, and CBA/J) [19]. However, the role of IL-4i1 in host immunity to *Mtb* infection has not been investigated to date.

In the current study, we provide evidence of the role of IL-4i1 in regulating macrophage-related immune responses during the early and late stages of *Mtb* infection. Chemical blocking of IL-4i1 enzymatic activity with aromatic blockers and IL-4i1 deletion in vitro reduced the *Mtb* colony-forming unit (CFU) burden in macrophages. IL-4i1 deficiency rendered mice more protective against acute and chronic *Mtb* infection with modulated responses in myeloid cells, which could promote a protective proinflammatory tissue environment.

## MATERIALS AND METHODS

### Generation of Bone Marrow–Derived Macrophages, Monocyte-Derived Macrophages, Benzoic Acid Treatment, *Mtb* Infection, and Cell Viability Assays

Bone marrow–derived macrophages (BMDMs) were generated from 8–12-week-old BALB/c mice, as described elsewhere [5]. After 10 days of differentiation, BMDMs were cultured in triplicates overnight for adherence to 96-well plates (Nunc) at 5 × 10^4^ cells per well. Monocyte-derived macrophages were isolated from human peripheral blood mononuclear cells obtained from healthy donors, as described elsewhere [3]. At 24 hours after adherence, BMDMs were either left unstimulated or stimulated with 100 U/mL of recombinant mouse IFN-γ or 100 U/mL of both IL-4 and IL-13 (BD Biosciences). 

For IL-4i1 enzymatic blocking experiments, BMDMs were simultaneously treated with IL-4 and dimethyl sulfoxide vehicle control (0.25%) or 2, 10, and 50 µmol/L of benzoic acid (Sigma Aldrich). A single-cell suspension of H37Rv and HN878 *Mtb* from frozen stock was prepared in Dulbecco’s modified Eagle medium supplemented with 10% fetal calf serum (Gibco; Thermo Fischer Scientific). After 24 hours of stimulation of BMDMs, or 24 hours after adherence of monocyte-derived macrophages, cells were infected with H37Rv or HN878 *Mtb* at a multiplicity of infection of 5. Cells were then washed once with culture medium to remove extracellular bacteria at 4 hours after infection and lysed at indicated time points with 0.1% Triton X-100 in phosphate-buffered saline solution. Lysates were plated on 7H10 agar plates containing 10% oleic acid-albumin-dextrose-catalase (OADC) and 0.5% glycerol and incubated at 37ºC for 14 days to the bacilli burden at different time points after infection. Cell viability was measured using the CellTiter-Blue assay (Promega).

### Histology

Lungs from *Mtb*-infected wild-type (WT) littermate controls and IL-4il–deficient (IL-4i1^−/−^) mice were isolated, fixed in neutral-buffered formalin. and stained with hematoxylin-eosin, as described elsewhere [4]. Briefly, the percentage of lung involvement was defined as the ratio of tissue occupied area in whole lung sections to the total lung tissue area, using the area measurement tool in NIS Advanced Software with Nikon 90i microscope [4]. The percentage of inducible nitric oxide (NO) synthase (iNOS) represents a blinded quantification of iNOS-positive pixel area relative to total tissue as a measure of protein expression.

### Statistical Analysis

All data were analyzed using GraphPad Prism 7.0 software and Student *t* tests (2 tailed with equal variance). Differences were considered significant at *P* <.05. 

## RESULTS

### Up-regulation of IL-4i1 Expression in *Mtb*-Infected M2 Macrophages and Promotion of Proinflammatory Responses by IL-4i1 Deficiency

**Figure 1.**
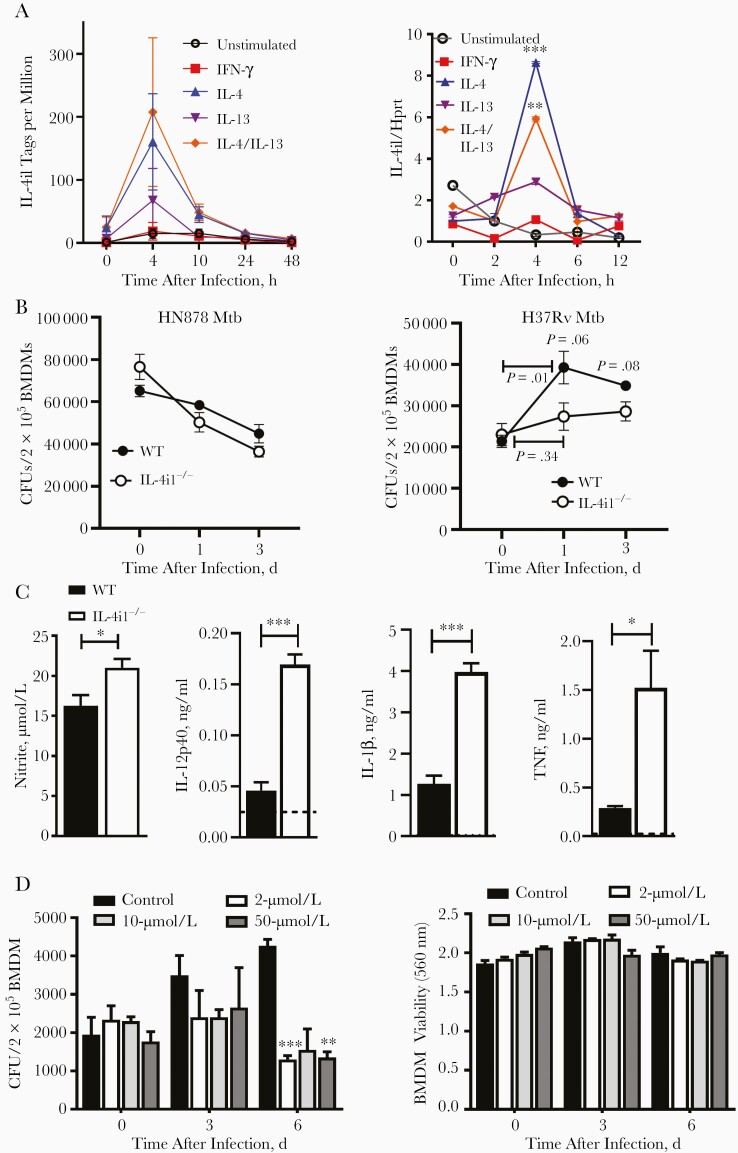
Interleukin 4i1 (IL-4i1) is highly expressed in HN878 *Mycobacterium tuberculosis* (*Mtb*)–infected interleukin 4 (IL-4)–stimulated macrophages and negatively regulates proinflammatory and bacterial killing effector functions. *A,* Bone marrow–derived macrophages (BMDMs) were generated from BALB/c mice and stimulated overnight with 100 U/mL of interferon (IFN) γ, IL-4, or IL-4/interleukin 13 (IL-13). Subsequently, cells were infected with HN878 *Mtb* strain at a multiplicity of infection of 1:5. The time-course expression of IL-4i1 (tags per million) transcripts after *Mtb* infection was determined using CAGE (Cap Analysis of Gene Expression) sequencing, and messenger RNA expression of IL-4i1 normalized to hypoxanthine guanine phosphoribosyl transferase (Hprt) was determined with quantitative polymerase chain reaction. *B,* Intracellular *Mtb* growth in wild-type (WT) and IL-4il–deficient (IL-4i1^−/−^) BMDMs, measured 4 hours, 1 day, and 3 days after infection with H37Rv and HN878 *Mtb* strains. Abbreviation: CFUs, colony-forming units. *C,* Nitrite and cytokine production from *Mtb*-infected IL-4i1^−/−^ and WT BMDMs at 3 days after infection. Abbreviations: IL-1β, interleukin 1β; IL-12p40, interleukin 12p40; TNF, tumor necrosis factor. *D,* CFU burden and cell viability in IL-4–stimulated BMDMs treated with 2-, 10-, and 50-μmol/L benzoic acid (BzA) at the indicated time points; 0 days corresponds to 4 hours after infection. Data are representative of 2 independent experiments, and error bars denote means with standard errors of the mean (n=3–4). ∗*P*<.05; ∗∗*P*<.01; ∗∗∗*P*<.001 (2-tailed Student *t* test).

Because IL-4i1 is mainly expressed in APCs, while serving a role in macrophage polarization, and has been reported to have antimicrobial properties [13, 15, 17], we investigated the expression kinetics of IL-4i1 and its role in *Mtb*-infected differentially polarized BMDMs. CAGE transcriptomics showed up-regulated expression of IL-4i1 in IL-4- or IL-4/IL-13–prestimulated BMDMs 4 hours after infection with HN878 *Mtb* (Figure 1A). We then used quantitative polymerase chain reaction to confirm significantly induced IL-4i1 expression in M2 (IL-4 or IL-4/IL-13) polarized *Mtb*-infected macrophages (Figure 1A). Human IL-4i1 expression was also significantly increased after HN878 *Mtb* infection in human monocyte–derived macrophages (Supplementary Figure 1*A*). IL-4i1 deficiency in BMDMs resulted in a decreasing trend of intracellular mycobacterial growth at 1 and 3 days after infection with virulent H37Rv *Mtb* strain (Figure 1B). 

To further investigate the decreased mycobacterial burden, IL-4i1^−/−^ BMDMs infected with H37Rv *Mtb* strain displayed significant up-regulated *Nos2* mRNA expression compared with WT BMDMs at 4 and 24 hours after infection (Supplementary Figure 1*B*), suggesting polarization toward restrictive phenotype. *Nos2* mRNA expression was complemented by an increase in nitrite production in IL-4i1^−/−^ BMDMs at 3 days after infection, which was correlated with a decreasing trend in intracellular *Mtb* growth) and a significant increase in interleukin 12p40 (IL-12p40), interleukin 1β (IL-1β), and tumor necrosis factor (TNF) levels (Figure 1C). To investigate the functional role of IL-4i1 during *Mtb* infection in macrophages, we inhibited IL-4i1 enzymatic activity using an aromatic competitor, benzoic acid, as described elsewhere [14, 20, 21]. BMDMs were treated with different concentrations of benzoic acid and IL-4 stimulation, followed by H37Rv *Mtb* infection to determine the mycobacterial burden. At 6 days after infection, the CFU burden was significantly reduced in benzoic-acid treated BMDMs compared with control, without inducing cell cytotoxicity (Figure 1D). The product of IL-4i1 enzymatic activity, H_2_O_2_, has been shown to inhibit NO synthase 2 (*Nos2*) gene expression [22]. 

Supplementation of H_2_O_2_ reversed the nitrite levels in IL-4i1^−/−^ BMDMs to WT BMDMs levels in supernatants collected at 3 days after *Mtb* infection (Supplementary Figure 1*C*), suggesting that modulation of NO levels by IL-4i1 is mediated through H_2_O_2_. However, H_2_O_2_ supplementation did not inhibit the antimycobacterial effects of benzoic acid, suggesting that benzoic acid–mediated bacterial killing does not depend solely on increased NO levels (Supplementary Figure 1*D*). Because IL-4i1 catalyzes phenylalanine metabolism, we investigated whether externally administered phenylalanine levels can result in increased antibacterial activity in IL-4i1^−/−^ BMDMs [23]. We observed an increasing trend of phenylalanine levels intracellularly in BMDMs during *Mtb* infection, and these levels were significantly increased in IL-4i1^−/−^ BMDMs compared with WT BMDMs 1 day after infection (Supplementary Figure 1*E*). 

Interestingly, supplementation of WT BMDMs with phenylalanine and the metabolic product of IL-4i1 catabolic activity, phenylpyruvate, decreased the intracellular *Mtb* burden, indicating antimycobacterial properties of phenylalanine metabolism without significantly affecting nitrite levels (Supplementary Figure 1*F* and 1*G*). Dendritic cells are also strong producers of IL-4i1 enzyme. Hence, we next investigated similar immunomodulatory and antibacterial properties in bone marrow–derived dendritic cells (BMDCs) [24]. The IL-4i1^−/−^ BMDCs showed significantly decreased intracellular growth of *Mtb* with concomitant increased nitrite and IL-1β levels, similar to the results obtained in BMDMs, while TNF and IL-12p40 levels remained unchanged (Supplementary Figure 1*H*–1*M*)_._ Taken together, these findings suggest an important role for IL-4i1 in modulating APC polarization and antimycobacterial activity during *Mtb* infection.

### Increased Protection Against Acute H37Rv *Mtb* Infection in IL-4i1^−/−^ Mice, With Reduced Mycobacterial Burden and Lung Histopathology

We next investigated the role of IL-4i1 during acute *Mtb* infection in vivo. We first performed polymerase chain reaction of tail biopsy specimens from IL-4i1^−/−^ mice to confirm the deletion; the mutant allele had an amplicon of 187 base pairs, compared with 325 base pairs for the WT allele amplicon (Supplementary Figure 2*A*). We further confirmed the absence of IL-4i1 protein in the splenocytes of IL-4i1^−/−^ mice by flow cytometry in CD3^+^CD4^+^ T cells, CD3^+^CD8^+^ T cells, CD19^+^ B cells, CD11b^+^F4/80^+^ macrophages, and CD11b^+^CD11c^+^ dendritic cells. The fluorescence levels in IL-4i1^−/−^ mice were comparable to those in the isotype control, as indicated by the geometric mean fluorescence intensity in the histogram plots (Supplementary Figure 2*B* and 2*C*). Although other cell types also express IL-4i1, macrophages and dendritic cells showed the highest expression of IL-4i1 compared with B- and T lymphocytes in splenocytes from control littermates (Supplementary Figure 2*D* and 2*E*). 

**Figure 2.**
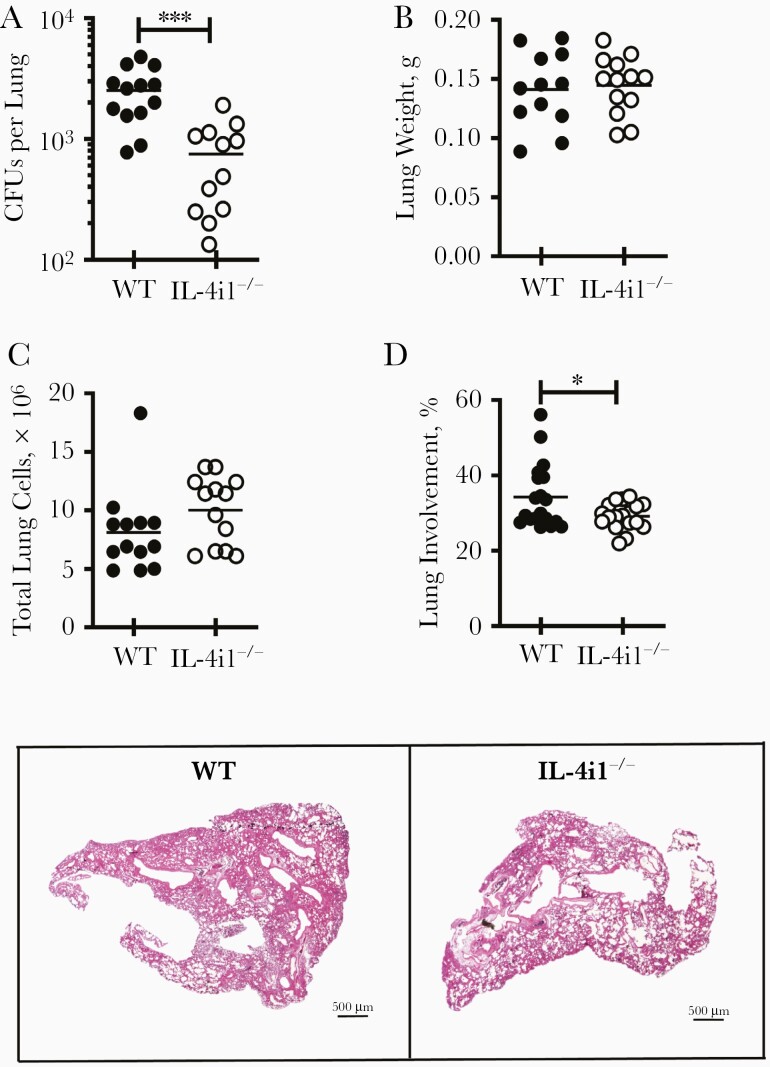
Interleukin 4il (IL-4i1)–deficient (IL-4i1^−/−^) mice displayed increased protection against acute H37Rv *Mycobacterium tuberculosis* (*Mtb*) infection with reduced pathology. *A,* Control littermate (wild-type [WT]) and IL-4i1^−/−^ mice were infected intranasally with 100 colony-forming units (CFUs) of H37Rv *Mtb* strain per mouse (n=5–8 per group). The mycobacterial burden in the lungs was measured by CFU count 21 days after infection. *B,* Lung weights were measured in H37Rv-infected mice 3 weeks after infection*. C,* Total cell numbers from single-cell suspensions of *Mtb*-infected lung tissues. *D,* Lung lesions were quantified by measuring the area of solid tissue versus ventilated air spaces from 3 deep-cut hematoxylin-eosin–stained lung sections per mouse (30 μm apart) and representative histopathological sections (original magnification ×20). Data in *A–C* are shown as the pool of 2 independent experiments, and data in *D* are representative of 2 independent experiments. ∗*P*<.05; ∗∗∗*P*<.001 (Student *t* test).

To determine the consequence of IL-4i1 deletion in *Mtb* infection in vivo, control littermate (WT) and IL-4i1^−/−^ mice were infected intranasally with 100 CFUs of H37Rv *Mtb* strain, and mycobacterial burdens (CFUs counts) were measured 21 days after infection in lungs of infected mice. Mycobacterial loads were significantly reduced in IL-4i1^−/−^ mice compared with WT mice (Figure 2A). Lung weights and total lung cell numbers were not affected in the absence of IL-4i1 (Figure 2B and 2C). However, pulmonary histopathological analysis showed significantly reduced lesion sizes in IL-4i1^−/−^ mice compared with WT mice (Figure 2D).

**Figure 3.**
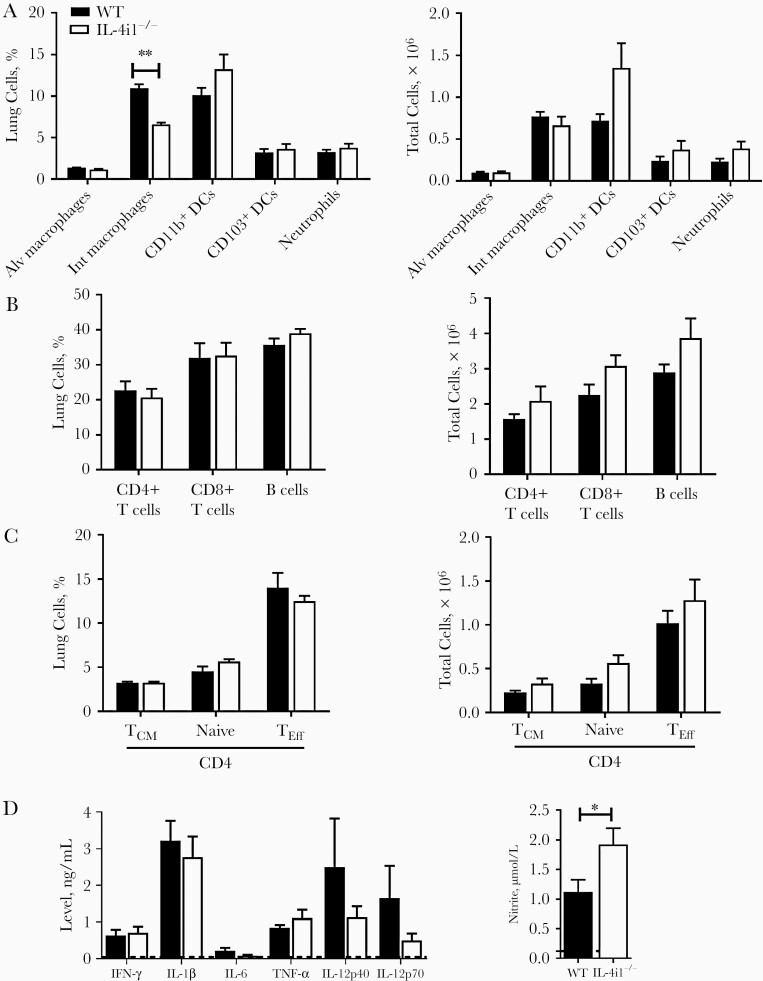
Interleukin 4 il (IL-4i1) deletion in mice reduces interstitial macrophage recruitment in H37Rv *Mycobacterium tuberculosis* (*Mtb*)–infected lungs, increases nitric oxide production, but does not change T-cell numbers. *A,* Control littermate (wild-type [WT]) and IL-4i1–deficient mice (IL-4i1^−/−^) were infected with 100 colony-forming units (CFUs) of H37Rv *Mtb* strain (n=5 per group). Percentage and total cell numbers of CD11c^+^SiglecF^+^ alveolar (Alv) and CD11b^+^F4/80^+^ interstitial (Int) macrophages, CD11b^+^CD11c^+^MHCII^+^ and CD11c^+^CD103^+^MHCII^+^ dendritic cells (DCs), and CD11b^+^Ly6G^+^ neutrophils 21 days after infection. *B,* Percentage and total cell numbers of CD19^+^ B cells, CD3^+^CD4^+^ T cells and CD3^+^CD8^+^ T cells in the lungs of infected mice. *C,* Percentage and total cell numbers of CD4^+^CD44^−^CD62L^+^ naive T cells, CD4^+^CD44^+^CD62L^−^ effector/effector memory T cells (T_eff_), and CD4^+^CD44^+^CD62L^+^ central memory T cells (T_CM_). *D,* interferon (IFN) γ, interleukin 1β (IL-1β), interleukin 6 (IL-6), tumor necrosis factor (TNF) α, interleukin 12p40 (IL-12p40), interleukin 12p70 (IL-12p70), and nitrite levels in lung homogenates. Dashed line indicates the assay’s limit of detection. Data are representatives of 2 independent experiments; error bars denote means with standard errors of the mean.∗*P*<.05; ∗∗*P*<.01 (Student *t* test).

IL-4i1 is largely characterized as serving a functional role in T-cell activation and proliferation and, reported more recently, B-cell activation [12, 13, 16]. To determine cellular infiltration in the lungs, we used flow cytometry to determine different innate and adaptive immune cell populations in the lungs 21 days after infection with H37Rv *Mtb*. IL-4i1 deletion significantly reduced interstitial macrophage percentages, but no differences were observed between *Mtb*-infected WT and IL-4i1^−/−^ mice in conventional CD11b^+^ and CD103^+^ dendritic cells and neutrophil recruitment (Figure 3A). There were also no differences in the frequencies of B cells and different T-cell subsets, nor in their proliferative status, marked by the comparable expressions of Ki67 in WT and IL-4i1^−/−^ mice (Figure 3B and 3C and Supplementary Figure 3*A* and 3*B*). Proinflammatory cytokine production, including IFN-γ and IL-12 levels, was not affected in lung homogenates from either group, except for a significant increase in NO production 21 days after infection in IL-4i1^−/−^ compared with WT mice (Figure 3D). Collectively, these data suggest that IL-4i1 deletion renders mice more protected against acute H37Rv *Mtb* infection, with concomitant reduction in pulmonary pathology, decrease in mycobacterial burden and increase in lung nitrite levels.

### Effect of IL-4i1 Deletion in HN878 *Mtb* Infection, With Promotion of “M1-like” Restrictive Macrophage Numbers at 12 days Post-infection

**Figure 4.**
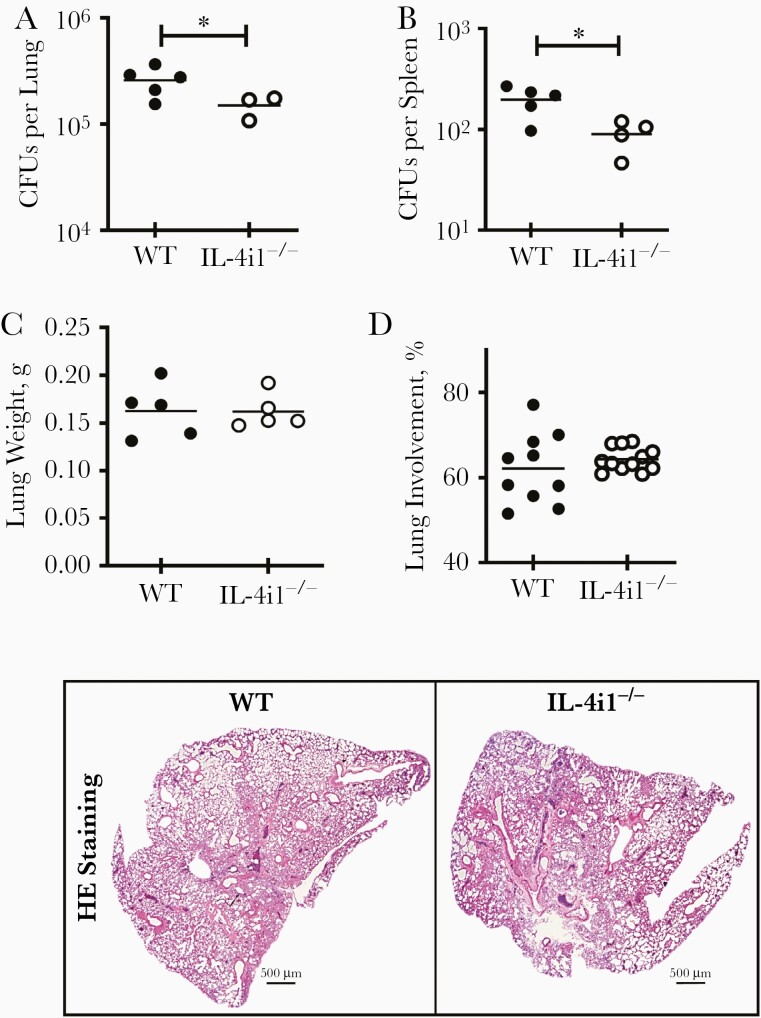
Interleukin 4i1 (IL-4i1)–deficient (IL-4i1^−/−^) mice were resistant to HN878 *Mycobacterium tuberculosis* (*Mtb*) infection at 3 weeks. *A,* Control littermate (wild-type [WT]) and IL-4i1^−/−^ mice were infected intranasally with 100 colony-forming units (CFUs) of hypervirulent HN878 *Mtb* strain (n=3–5 per group). Mice were euthanized 21 days after infection to measure mycobacterial burden in the lungs. *B,* Spleen bacterial burdens in HN878-infected mice. *C,* Lung weights were measured in HN878-infected mice 3 weeks after infection. *D,* Tissue involvement was quantified from 2–3 deep-cut hematoxylin-eosin (HE)–stained lung sections per mouse (30 μm apart) and representative histopathological sections (original magnification ×20). Error bars denote means with standard errors of the mean. ∗*P*<.05 (Student *t* test).

To determine the role of IL-4i1 in macrophage-mediated immune responses against *Mtb* infection, we intranasally infected WT and IL-4i1^−/−^ mice with hypervirulent HN878 *Mtb* (100 CFUs per mouse), euthanized them 21 days after infection. IL-4i1 deletion significantly reduced mycobacterial burden in the lungs (Figure 4A) and spleen (Figure 4B), while lung weights were not affected (Figure 4C). In contrast to the reduced histopathology observed in IL-4i1^−/−^ lung lesions 21 days after H37Rv *Mtb* infection (Figure 2D), the resulting lesion sizes after HN878 *Mtb* infection were comparable in both groups (Figure 4D), which could potentially be attributed to the minor 1.7-fold reduction in lung CFU counts in IL-4i1^−/−^ compared with WT mice (Figure 4A). As observed in H37Rv infection, effector CD4^+^CD44^+^ T-cell proliferation, as measured by Ki67 expression, was not affected in HN878 *Mtb* infection (Supplementary Figure 3*C*), pointing toward the prominent effect of IL-4i1 on myeloid cells rather than T-cell numbers in acute infection. Although IL-4i1 mRNA expression was significantly up-regulated in the lungs of WT mice 3 weeks after infection, compared with naive mice (Supplementary Figure 3*D*), the mRNA levels of other immunosuppressive metabolic enzymes, indoleamine 2,3-dioxygenase 1 and 2, remained unchanged in the lungs of WT and IL-4i1^−/−^ mice 3 weeks after HN878 *Mtb* infection (Supplementary Figure 3*E*).

**Figure 5.**
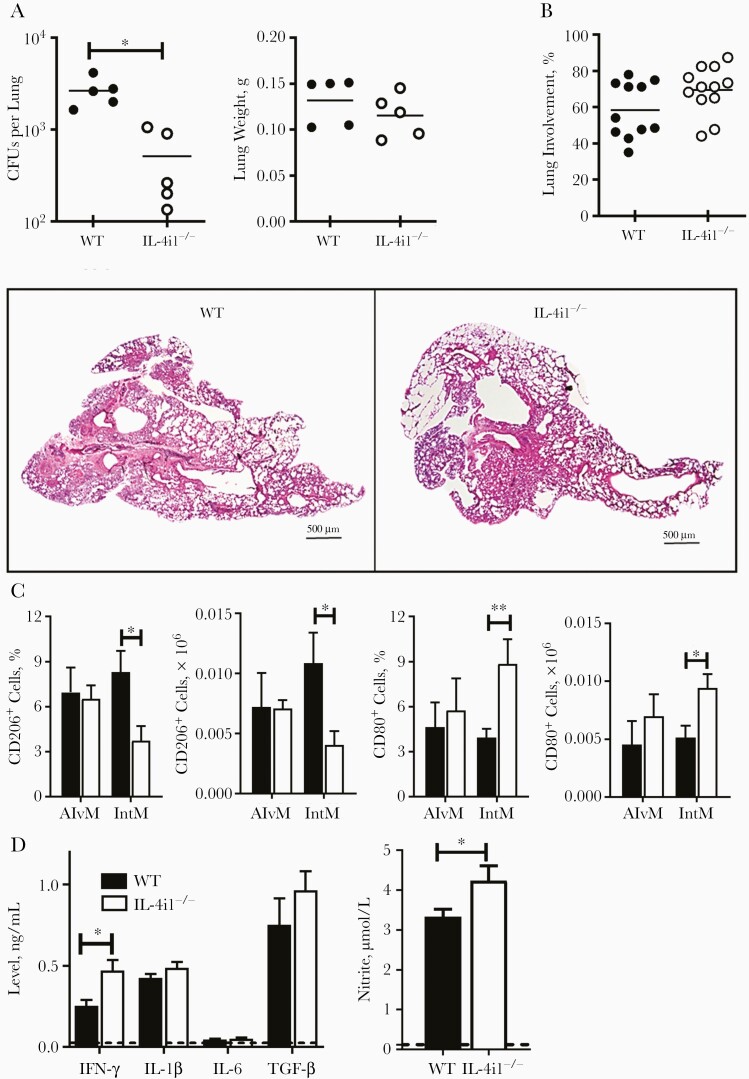
Interleukin 4i1 (IL-4i1) deficiency promotes the recruitment of “M1-like” restrictive macrophages and chemokine production in early HN878 *Mycobacterium tuberculosis* (*Mtb*) infection. *A,* Control littermate (wild-type [WT]) and IL-4i1–deficient (IL-4i1^−/−^) mice were infected intranasally with 100 colony-forming unit (CFUs) of hypervirulent HN878 *Mtb* strain (n=5 per group). Mice were euthanized 12 days after infection. Mycobacterial burden in the lungs and lung weights were measured. *B,* Lung tissue involvement was quantified from 2–3 deep-cut hematoxylin-eosin–stained lung sections per mouse (30 μm apart) and representative histopathological sections (original magnification ×20). *C,* Percentage and total cell numbers of CD206^+^ and CD80^+^ alveolar (Alv; CD11c^+^SiglecF^+^) and interstitial (Int; CD11b^+^F4/80^+^) macrophages in infected WT and IL-4i1^−/−^ mice. *D,* IFN-γ, interleukin 1β (IL-1β), interleukin 6 (IL-6), transforming growth factor (TGF) β and nitrite levels in lung homogenates of infected WT and IL-4i1^−/−^ mice. The dashed line indicates the limit of detection of the assay; error bars denote means with standard errors of the mean. ∗*P*<.05; ∗∗*P*<.01 (Student *t* test).

To determine the role of IL-4i1 on early antimycobacterial immune responses, we infected WT and IL-4i1^−/−^ mice intranasally with 100 CFUs of hypervirulent HN878 *Mtb* strain and euthanized them 12 days after infection. IL-4i1 deletion significantly reduced lung mycobacterial burden as early as 12 days after infection, while lung weights (Figure 5A), free alveolar air spaces, and lung tissue pathology were not affected by the absence of IL-4i1 (Figure 5B). Previously, IL-4i1 has been reported to promote macrophage polarization toward the M2 phenotype in vitro, through signal transducer and activator of transcription 6 phosphorylation and partly through its role in arginine depletion, promoting arginase production [17]. To determine the role of IL-4i1 in macrophage polarization in vivo, 12 days after infection, we used flow cytometry to determine the number of “M1-like” restrictive and “M2-like” permissive macrophages infiltrating the lungs of WT and IL-4i1^−/−^ mice. Interestingly, percentages and numbers of M2-like CD206^+^ interstitial macrophages were significantly reduced in IL-4i1^−/−^ compared with WT mice, and the percentages and numbers of M1-like CD80^+^ interstitial macrophages were significantly increased (Figure 5C). A significant increase in lung IFN-γ and nitrite production was observed in IL-4i1^−/−^ mice compared with WT mice (Figure 5D). Taken together, these data suggest that IL-4i1 deletion leads to increased proinflammatory macrophage-mediated immune responses that are characterized by a M1-like phenotype in the early stages of *Mtb* infection in vivo.

### Protective Phenotype of IL-4i1^−/−^ Mice With Chronic HN878 *Mtb* Infection

**Figure 6.**
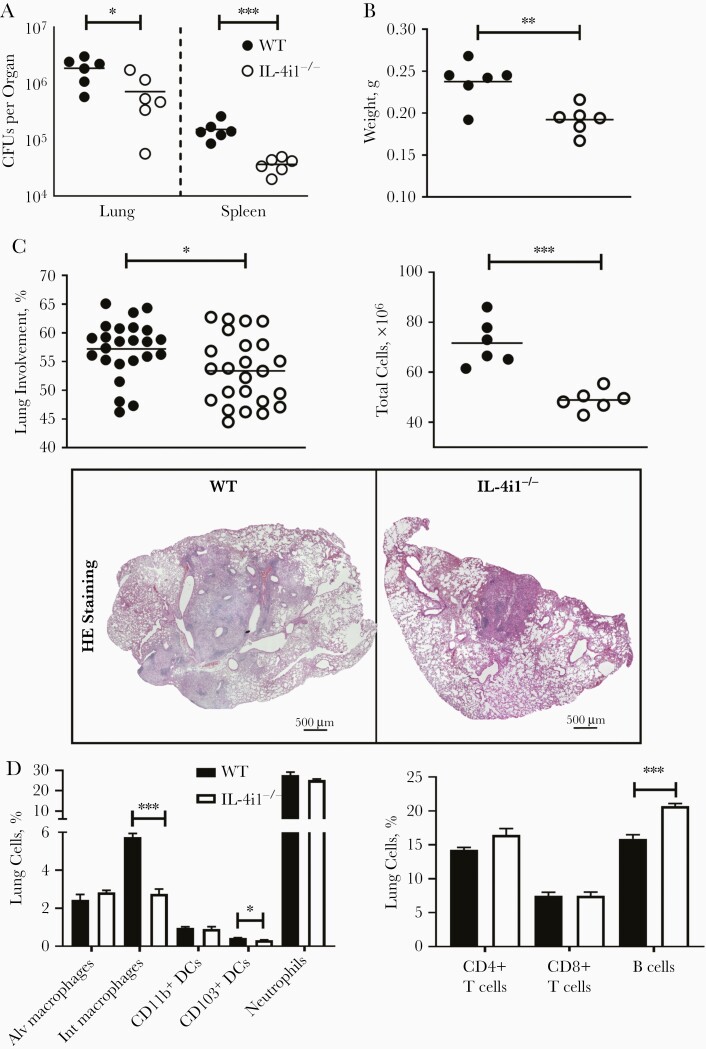
The decreased bacterial burdens in interleukin 4i1 (IL-4i1)–deficient (IL-4i1^−/−^) mice are maintained in chronic stages of HN878 *Mycobacterium tuberculosis* (*Mtb*) infection. *A,* Control littermate (wild-type [WT]) and IL-4i1^−/−^ mice were infected intranasally with 100 colony-forming units (CFUs) of HN878 *Mtb* strain per mouse (n=6 per group). Mice were euthanized 8 weeks after infection. Mycobacterial burdens in the lungs and spleen were measured by CFU assay. *B,* Total lung weight (*upper panel*) and total cell numbers from single-cell suspensions (*lower panel*) were quantified. *C,* Lung tissue involvement was quantified from 4 deep-cut hematoxylin-eosin (HE)–stained lung sections per mouse (30–45 μm apart) and representative histopathological sections (original magnification ×20). *D, Left,* Percentages of alveolar (Alv) and interstitial (Int) macrophages, CD11b^+^ and CD103^+^ dendritic cells (DCs), and neutrophils in live cells 8 weeks after infection. *Right,* Percentages of B cells, CD4^+^ T cells, and CD8^+^ T cells in live cells. Detailed gating strategy can be seen in Supplementary Figure 5. Data shown are from a single experiment; error bars denote means with standard errors of the mean.∗*P*<.05; ∗∗*P*<.01; ∗∗∗*P*<.001 (Student *t* test).

We investigated whether the early acquired protective phenotype of IL-4i1^−/−^ is still maintained in the chronic stages of the infection. WT and IL-4i1^−/−^ mice were infected with 100 CFUs of hypervirulent HN878 *Mtb* and euthanized 8 weeks after infection. IL-4i1^−/−^ mice still displayed significant reduced mycobacterial burdens in the lungs and spleen (Figure 6A). The decreased mycobacterial burdens were accompanied by significantly decreased lung weights and reduced total lung cell numbers in IL-4i1^−/−^ mice (Figure 6B). In addition, the percentage of lung involvement was significantly reduced in IL-4i1^−/−^ lung sections, compared with WT lungs (Figure 6C). Interestingly, was observed in the acute stages of *Mtb* infection, IL-4i1^−/−^ mice exhibited a significantly reduced percentage of interstitial macrophages and CD103^+^ conventional dendritic cells (cDC1) cells with distinctly increased B-cell frequencies (Figure 6D). 

We also explored the macrophage M1/M2 polarization state at the chronic stage of infection, considering that the decreased mycobacterial burdens could affect macrophage activation. As similarly shown in the acute *Mtb* infection, M2-like CD206^+^ interstitial macrophages were significantly reduced in IL-4i1^−/−^ compared with WT mice, but the percentages of M1-like CD80^+^ interstitial macrophages were significantly decreased (Supplementary Figure 3*F* and 3*G*). IL-4i1^−/−^ mice also displayed significant increased percentage of naive CD4^+^ T cells (Supplementary Figure 3*H*). 

In terms of cytokine responses, IL-4i1^−/−^ mice exhibited significantly reduced IL-1β levels in the lung homogenates, while other proinflammatory cytokines or nitrite levels remained unchanged (Supplementary Figure 3*I* and 3*J*). To understand the effect of IL-4i1 deficiency on cytokine gene expression in the specific cell subsets rather than the total cytokine levels in the lungs, we used flow cytometry to perform cell sorting of lung resident APCs (macrophages and cDCs), CD4^+^ and CD8^+^ T cells (Supplementary Figure 4*A*). Similar to results obtained from the in vitro *Mtb* infection in BMDCs (Supplementary Figure 1*J* and 1*K*), IL-4i1^−/−^ lung cDCs significantly expressed increased *Nos2* and *Il1b* when compared with WT lung cDCs (Supplementary Figure 4*B*–4*G*). *Tnf* and *Il1b* mRNA expression levels were also significantly increased in cell-sorted lung CD4^+^ T cells from IL-4i1^−/−^ mice, compared with WT mice. Taken together, the deletion of the *Il4i1* gene rendered mice more protected against *Mtb* during the chronic stage of infection, with consistent effects on mycobacterial burdens and diminished tissue damage.

## DISCUSSION

Classically activated macrophages are characterized by the release of NO, which is essential for *Mtb* killing [2]. One immune evasion mechanism *Mtb* uses is to alter the transcriptional landscape of macrophages to promote alternative activation, inducing an M2-like environment [4–8]. Our group has previously reported that *Mtb* induces arginase production in macrophages, independent of the IL-4Ra signaling pathway [4]. To determine the functional role of IL-4i1 in macrophage polarization, Yue et al [17] showed that the IL-4i1 depletion in macrophages enhanced the expression of M1 markers, such as *Tnf, Il1b, Il12b,* and *Nos2* [17]. 

Similarly, we also showed that IL-4i1^−/−^ BMDMs were polarized toward an *Mtb*-restrictive phenotype marked by the increased mRNA expression of *Nos2* and increased NO, IL-12p40, IL-1β, and TNF production. In line with BMDM data, BMDC experiments also showed that IL-4i1^−/−^ cells have increased bacterial killing ability, with augmented NO and IL-1β expression suggesting that these antibacterial players might be responsible for *Mtb* killing in IL-4i1 deficiency. IL-4i1 is preferentially localized in the lysosome, where it is more active in an acidic pH environment that allows the enzyme’s activity to lead to the production of H_2_O_2_ and ammonia, contributing to a hostile environment for bacterial growth [13–15]. 

Boulland et al [13] showed that colocalization between lysosome and cytoplasm confers an increased enzymatic activity and antibacterial properties. However, in the current study, the reduced mycobacterial burden was a consequence of IL-4i1 depletion, suggesting that the absence of IL-4i1 antibacterial properties was compensated by other mycobacterial killing effector mechanisms, such as increased NO levels. In addition, accumulating phenylalanine in BMDMs in the absence of IL-4i1 can result in decreased bacterial burdens, as we have shown that both phenylalanine and phenylpyruvate supplementation can increase bacterial killing in WT and IL-4i1^−/−^ BMDMs, pointing out that the antibacterial activity of phenylalanine does not depend on IL-4i1.

To determine the role of IL-4i1 in potentially regulating host immune responses during mycobacterial infection in vivo, we infected WT and IL-4i1^−/−^ mice with H37Rv and HN878 *Mtb* strains. IL-4i1 deficiency led to improved protection of mice against acute H37Rv and acute/chronic HN878 *Mtb* infection, as denoted by a reduced mycobacterial burden for both strains and slightly reduced histopathology at later stages of infection. It is possible that reduced histopathology is due to decreased bacterial burdens rather than to direct effects of IL-4i1 deficiency on lung inflammation. Decreased cellular recruitment and lung tissue involvement were more noticeable in chronic stages, with consistently decreased interstitial macrophage numbers.

In the current study, T- and B-cell percentages and cell numbers were not affected in the absence of IL-4i1 during acute *Mtb* infection. We observed comparable populations of B cells, effector T cells, regulatory T-reg, and Ki67^+^ proliferating T cells in WT and IL-4i1^−/−^ mice. Interestingly, B-cell frequencies in chronic infection were increased distinctively, in line with previous observations that show IL-4i1 deficiency induces B-cell egress from bone marrow [12]. The chronic antigenic stimulation in the later stages of *Mtb* infection may have exaggerated dysregulation in B-cell maturation and recruitment to peripheral sites in IL-4i1^−/−^ mice. Our findings demonstrated an innate immune regulatory role for IL-4i1 during the early acute phase of *Mtb* infection; however, the role of IL-4i1 in the chronic phase of *Mtb* infection with the focus on B cells still needs to be investigated in future studies.

To determine the role of IL-4i1 on early immune responses against *Mtb* infection, we infected mice with HN878 *Mtb* for 12 days. The 12-day time point was selected because the early innate immunity is well established and the mycobacterial load is similarly distributed between interstitial and alveolar macrophages [25]. Bacterial burden was reduced as early as 12 days after infection in IL-4i1^−/−^ mice. We further observed a reduction in MHCII^+^CD206^+^ permissive macrophages and an increase in M1-like MHCII^+^CD80^+^ restrictive macrophages in IL-4i1^−/−^ mice. Increased restrictive macrophage population was associated with an increase in pulmonary IFN-γ and NO production, suggesting that IL-4i1 regulates macrophage-mediated inflammatory responses. These findings are in line with those of Psachoulia et al [26], who reported that IL-4i1 modulates inflammation by reducing IFN-γ expression in central nervous system lesions and splenocytes. Furthermore, IL-4i1^−/−^ mice exhibited higher numbers of CD11b^+^iNOS^+^ macrophages in central nervous system lesions [26]. In conclusion, our findings suggested that, in early infection, increased expression of IL-4i1 regulates APC-mediated immunity. These findings provide an indication of the immune regulatory role of IL-4i1 during early *Mtb* infection.

## Supplementary Data

Supplementary materials are available at *The Journal of Infectious Diseases* online. Supplementary materials consist of data provided by the author that are published to benefit the reader. The posted materials are not copyedited. The contents of all supplementary data are the sole responsibility of the authors. Questions or messages regarding errors should be addressed to the author.

jiab558_suppl_Supplementary_Material

## Notes

***Acknowledgments.*** We thank the University of Cape Town Research Animal Facility for maintaining the mice; Munadia Ansari for genotyping the mice; and Zarinah Sonday, Faried Abbass, Marlon Peterson, for their valuable technical assistance. We are also grateful to Lizette Fick for her excellent histology services.

***Financial support.*** This work was supported by the South African Medical Research Council (SAMRC) and its Division of Research Capacity Development under the SAMRC Internship Scholarship Programme (from funding received from the South African National Treasury to L. H.; SAMRC Intercalated Bursary to S.R.N.S.); the International Centre for Genetic Engineering & Biotechnology (Arturo Falaschi fellowships from the Cape Town Component to M. O. and J. E. C. and support to F. B.); the Ministry of Education, Culture, Sports, Science and Technology of the Japanese government (research grant for the Special Coordination Funds for Promoting Science and Technology to H. S.); the National Research Foundation (NRF)/Department of Science and Technology (DST)–South African Research Chair Initiative, NRF Competitive Programme for Unrated Researchers; the DST/NRF Collaborative Postgraduate Training Programme; the Brazil, Russia, India, China and South Africa (BRICS) Multilateral Joint Science and Technology Research Collaboration (grant 110482 to R. G.); and the Wellcome Centre for Infectious Diseases Research in Africa (203135Z/16/Z fund supporting biosafety level 3 facilities and equipment).

***Potential conflicts of interest.*** authors: No reported conflicts. All authors have submitted the ICMJE Form for Disclosure of Potential Conflicts of Interest. Conflicts that the editors consider relevant to the content of the manuscript have been disclosed.

Presented in part: Sixth South African Immunology Society Conference 3–6 September 2017, Gordon’s Bay, South Africa Integrating Metabolism and Immunity (E4) Keystone Symposium, 29 May–2 June 2017, Dublin, Ireland 50th Union World Conference on Lung Health, 30 October–2 November 2019, Hyderabad, India Early Career Scientist Convention SAMRC, 9–11 October 2019, Cape Town, South Africa.

Interleukin 4i1 (IL-4i1) regulates antigen-presenting cell–associated host immune response during early and chronic stages of *Mycobacterium tuberculosis* (*Mtb*) infection. IL-4i1 deletion in mice improved protection against *Mtb* through reduction of mycobacterial burden and improved type 1 effector immune responses.
