# Supplementary material for: IL-4i1 Regulation of Immune Protection During Mycobacterium tuberculosis Infection
Source: J Infect Dis. 2021 Nov 5;224(12):2170–80. doi: 10.1093/infdis/jiab558 (PMC8672763; doi:10.1093/infdis/jiab558)
Supplement: jiab558_suppl_Supplementary_Material [file jiab558_suppl_supplementary_material.docx]

**Supplementary Methods**

**Mice**

Mice used in the study were resuscitated from B6;129S5-*Il4i1^tm1Lex^*/Mmucd, identification number 011726-UCD, obtained from the Mutant Mouse Regional Resource Center (MMRRC), an NIH funded strain repository donated by Lexicon Genetics, Inc. F2 generation was obtained from MMRRC and backcrossed for 9 generations to wild-type BALB/c background. All experimental mice aged 8-12 weeks, were both sex and age-matched. Mice were kept under pathogen-free conditions in individually ventilated cages at the University of Cape Town’s animal facility unit Biosafety level 3 (BSL-3). All experimental procedures were carried following the South African National Standard (SANS 10386:2008). The protocols (AEC: 015/036 and AEC: 015/040) were approved by the Ethics Committee, University of Cape Town.

**Genotyping and confirmation of IL-4i1 deletion**

DNA from tail biopsies and splenocytes of naive IL-4i1^-/-^ mice and control littermates were isolated for confirmation of IL-4i1 deletion with the following primer sequences: Mutant forward 5’- GCAGCGATCGCCTTCTATC-3’ and reverse 5’-GTGCTCACTTCCTCTTTGCGACT-3’ and Wild-type primers: forward 5’-TTGAGACCTTTCTTTCCGAGCAG-3’ and reverse 5’-AGGCTAAACCTTG-3. The PCR products were separated by electrophoresis on SYBR Safe (Thermo Fisher) stained agarose gel, followed by visualization under UV light. The amplicon fragment for the wild-type allele is 325 bp and 187bp for the IL-4i1 mutant allele.

**Bone marrow-derived dendritic cell (BMDC) Generation**

BMDC were generated from bone marrow cells of 8-12 weeks old WT and IL-4i1 KO mice. Briefly, after flushing bone marrow cells and making single-cell suspension, bone marrow cells were seeded in 90 mm bacterial grade Petri dishes (ThermoFisher Scientific) in 10 ml R10 media (RPMI+10%FCS+1%Penicillin/Streptomycin+2 mM L-Glutamine+50 µM β-Mercaptoetanol+50% GM-CSF Conditioned Media which is supernatant from J558L cell lines). On day 3, additional 10 ml media was added to Petri dishes and on day 6, 10 ml media was removed, briefly centrifuged at 400xg for 5 min, the pellet was resuspended with fresh 10 ml R10 media and transferred back to Petri dishes. On day 8, the loosely adherent cells were collected as these cells were shown to exhibit dendritic cell phenotype (Data not shown). Mtb infections were performed in BMDC for downstream assays.

**RNA extraction and qPCR**

BMDM and mouse lungs were lysed in 1 ml of Qiazol (Qiagen) and total RNA was extracted by RNeasy Mini kit (Qiagen). Sorted cells were lysed in 0.3ml of RLT Lysis Buffer (Qiagen) and RNA extracted with RNeasy Micro Kit (Qiagen). Total RNA was transcribed into cDNA using Transcriptor First Strand cDNA Synthesis Kit (Roche) according to the manufacturer’s instructions. qPCR was performed with LightCycler® 480 SYBR Green I Master mix in LightCycler® 480 II (Roche). Quantitative expression analysis of Il4i1, Nos2, Ido1, Ido2, Ifng, Il1b, Tnf, Il12b and Il6 were normalized against the housekeeping gene Hprt with primers. Primer sequences are provided in Suppl Table 1.

**Flow cytometry**

Left lobes of mouse lungs were dissected out aseptically and cut into small pieces that were followed by incubation in lung digestion buffer containing Dulbecco’s Modified Eagle Media (DMEM), 0.18 mg/mL Collagenase Type I (Sigma Aldrich) and 0.02 mg/mL DNase I (Sigma) for 1 hour at 37 ^o^C under constant rotation. The digested lung tissue was mechanically passed through 100 µm and 70 µm cell strainers sequentially. Red blood cells were lysed with RBC lysis buffer (155 mM NH4Cl, 12 mM NaHCO3, 0.1 mM EDTA). Cells were counted with the Trypan Blue exclusion test. Single-cell suspensions from lung tissues were stained for surface markers with the antibodies provided in Supplementary Methods. For surface staining, cells (1x10^6^) were labelled and washed with FACS buffer. After surface staining, cells were fixed with 2% paraformaldehyde overnight and transferred out of Biosafety Level 3 (BSL3) facility. Downstream staining and flow cytometry acquisition was performed in the BSL2 laboratory. For intracellular staining, BD Pharmingen Transcription Buffer Set (BD Biosciences) was used as per the manufacturer’s instructions to detect Foxp3 and Ki67 expression. The acquisition was conducted using BD LSR Fortessa, and data analysis was performed with FlowJo V9 software (Treestar, Ashland, OR, US). The following antibodies were used for flow cytometry staining. F4/80 (Clone BM8 PE-Cy7) from eBiosciences; CD206 (Clone C068C2 FITC) from BioLegend; CD4 (Clone RM4-5 PerCP-Cy 5.5 or PE or FITC), CD11c (Clone HL3 A700 or APC), CD11b (Clone M1/70 PerCP-Cy 5.5), CD64 (Clone X54-5/7.1 Pe-Cy7), Mer (Clone 108928 BV786), MHCII (Clone M5/114.15.2 A700), CD103 (Clone M290 PE), SiglecF (Clone E5-2440 APC-Cy7 or APC), CD44 (Clone IM7 FITC), CD62L (Clone MEL-14 V450), CD19 (Clone 1D3 PerCP-Cy5.5), CD8 (Clone 53-6.7 V500), LY6G (Clone 1A8 APC-Cy7); Foxp3 (Clone R16-71 APC), CD80 (Clone 16-10A1 V450), and Ki67 (PE) from BD Biosciences in FACS buffer (1X PBS with 1% BSA and 0.1% NaN3). Rabbit polyclonal IL-4i1 antibody and goat anti-rabbit secondary IgG (PE) were purchased from Abcam.

**Measuring Cytokine and Metabolite Concentration**

Lung homogenates were used to determine tissue IL-12p40, IL-12p70, IL-6, IFN-γ, TGF-β (BD Biosciences) IL-1β (R&D Scientific) and TNF (BioLegend) using enzyme-linked immunosorbent assay. Phenylalanine levels are measured Phenylalanine Asay Kit (Abcam) according to manufacturer’s instructions. Nitrite levels are quantified with Griess Assay. Absorbance and fluorescence levels were measured in SpectraMax iD5 Multimode Reader (Molecular Devices). Cytokine and metabolite levels were analyzed using SoftMax Pro 6 and 4-parameter curve fitting for standard curve generation.

**Data deposition**

CAGE FANTOM5 transcriptomic data library was deposited on: http://fantom.gsc.riken.jp/5 and mouse macrophage Mtb infection data can be extracted by FANTOM5 Table Extraction Tool.

https:/fantom.gsc.riken.jp/5/tet/#!/search/mm9.cage_peak_phase1and2combined_tpm_ann_ddecode.osc.txt.gz

**Supplementary Figure Legends**

**Figure S1: IL4-i1 modulates APC responses *in vitro*.** *A,* Time-course IL-4i1 TPM transcripts from human monocyte-derived macrophages infected with HN878 Mtb by CAGE-Seq. *B,* Nos2 mRNA expression in IL-4i1^-/-^ and WT BMDM 4 and 24hrs post-infection of H37Rv Mtb strain. *C,* BMDM were infected with H37Rv Mtb as previously described. At one day post-infection, 10 µM H_2_O_2_ and DMSO vehicle control (0.1%) were added. To measure the effect of H_2_O_2_ on Nos2 enzyme activity, the supernatants were collected at 3 days post-infection and Griess Reagent assay was performed to measure nitrite levels. *D,* BMDM were incubated with DMSO vehicle control (0.1%) or 2 and 50 µM of benzoic acid overnight. The following day, BMDM were infected with H37Rv Mtb at MOI 5. 4 hours post-infection BMDM were washed and stimulated with IL-4 (100U/ml). At one day post-infection BMDM were stimulated with 10 µM H_2_O_2_ or DMSO vehicle control and intracellular Mtb burden was measured 2 days post H_2_O_2_ stimulation. *E,* Intracellular phenylalanine levels were measured in naïve WT and IL-4i1^-/-^ BMDM or H37Rv Mtb infected BMDM at 1 day and 3 days post-infection. *F,* WT and IL-4i1^-/-^ BMDM were infected with H37Rv Mtb for 4 hours and media was replaced with vehicle (0.1% DMSO), 5 µM phenylalanine (PHA) or 0.5 µM phenylpyruvate (PPA). Intracellular bacterial burden was measured at 3 days post-infection. *###* denotes P<0.001 in comparison of WT vehicle-treated BMDM vs WT PPA treated BMDM *G,* Nitrite levels were measured on PHA and PPA supplemented Mtb-infected BMDM at 3dpi. *H,*  BMDC were generated from bone marrow cells of WT and IL-4i1^-/-^ mice and infected with H37Rv strain similar to BMDM CFU assays. Bacterial burdens were quantified 4 hours, 1 day and 3 days post-infection. *I,* Intracellular Mtb burden of WT and IL-4i1^-/-^ BMDC when they were infected with HN878 Mtb strain. *J-M,* Nitrite, IL-1β, TNF, IL-12p40 production on H37Rv Mtb infected IL-4i1-/- and WT BMDM at 3 days post-infection. Data are representatives of 2 independent experiments. Error bars denote mean±SEM, n=4. Two-tailed student’s *t-test* **P* < 0.05; ***P* < 0.01, ****P* < 0.001 was used to determine significance.

**Figure S2: Genotyping of IL-4i1 deficient mice.** *A,* Deletion of IL-4i1 was confirmed from tail cuts of wild-type and IL-4i1 knockout mice. The amplicon fragment for wild-type is 325 bp and 187bp for IL-4i1^-/-^. *B,* Geometric mean fluorescence intensity of IL-4i1 in different cell types of spleens from IL-4i1^-/-^ and wild-type littermate control mice. *C,* Confirmation of IL-4i1 deletion by flow cytometry from different cell types CD3^+^CD4^+^ T-cells, CD3^+^CD8^+^ T-cells, CD19^+^ B-cells, CD11b^+^F4/80^+^ macrophages, CD11b^+^CD11c^+^ dendritic cells isolated from spleen of IL-4i1^-/-^ (grey) and wild-type littermate control mice (black), red line represents isotype control. *D,* Cell-type expression of IL-4i1 by flow cytometry in spleens of wild-type littermate controls, CD11b^+^CD11c^+^ dendritic cells, CD11b^+^F4/80^+^ macrophages, CD3^+^CD4^+^ T-cells, CD3^+^CD8^+^ T-cells, CD19^+^ B-cells. *E,* Geometric mean fluorescence intensity of IL-4i1 in different cell types. Data are representatives of two independent experiments with at least n= 4-6 mice per group. Error bars denote mean±SEM. Student *t-test* analysis **P* <0.05.

**Figure S3: IL-4i1 deletion in mice does not affect lymphocyte population to greater level during acute and chronic Mtb infection.** *A,* Control littermate (WT) and IL-4i1 deficient mice (IL-4i1^-/-^) were infected with 100 CFU of H37Rv Mtb strain (n=5/group). Mice were sacrificed at 21 days post-infection. Percentage CD4^+^ Foxp3^+^ regulatory T-cells in the lungs. *B,* Percentage of proliferating Ki67^+^CD4^+^CD44^+^ effector T-cells in the lungs of infected mice. *C,* Control littermate (WT) and IL-4i1 deficient mice (IL-4i1^-/-^) were infected intranasally with 100 CFU of hypervirulent HN878 Mtb strain (n=3-5/group). Mice were sacrificed at 21 days post-infection to measure the percentage of Ki67 proliferating CD4+ CD44^+^ effector T cells. *D-E,* ll4i1, Ido1 and Ido2 mRNA expression in total lung tissue by RT-qPCR normalized to Hprt. *F,* CD80 expression measured in lung resident macrophage population during chronic HN878 infection. The frequencies show the percentage of CD80^+^ cells among live cells. *G,* CD206 expression measured in lung resident macrophage population during chronic HN878 infection. The frequencies show the percentage of CD206^+^ cells among live cells. *H,* The frequencies of CD4^+^ T cell memory subtypes among total live cells during chronic HN878 Mtb infection. *I,* Nitrite levels measured in the cell-free homogenates of chronic HN878 Mtb infected lungs by Griess assay. *J,* Pro-inflammatory cytokine levels in the cell-free homogenates of chronic HN878 Mtb infected lungs. Data in S3A-S3E are representatives of 2 independent experiments. Data in S3F-S3J is from one experiment. Error bars denote mean±SEM. Student’s t-test **P* < 0.05, ** *P* <0.01.

**Figure S4: IL-4i1 deletion renders lung cDC responses towards more pro-inflammatory phenotype during chronic HN878 Mtb infection** *A,* Gating strategy for sorting lung immune cells. Live cell gating can be seen in Figure S5A. CD4^+^ T cells (CD3^+^CD4^+^CD19^-^), CD8^+^ T cells (CD3^+^CD8^+^CD19^-^), lung resident macrophages (CD64^+^MerTK^+^CD3^-^CD19^-^) and lung cDC (CD11c^+^MHCII^+^CD64^-^MerTK^-^CD3^-^CD19^-^) populations were sorted on BD FACSAria Fusion in biosafety level 3 lab and lysed in RNA lysis buffer for downstream qPCR application. *B-G,* Ifng (B), Il1b (C), Tnf (D), Il12b (E), Il6 (F) and Nos2 (G) expressions were quantified in four different immune cell populations by qPCR. Absolute quantification is performed and the expression levels are normalized against Hprt housekeeping gene. Data is from one experiment. Error bars denote mean±SEM. Student’s t-test **P* < 0.05, ** *P* <0.01.

**Figure S5: Gating strategies employed to measure lung immune subsets in acute and chronic Mtb infected mice** *A,* Gating strategy to identify lung myeloid populations in chronic HN878 infected mice. CD80 and CD206 gating is applied to both alveolar macrophage and interstitial macrophage populations; however only shown in interstitial macrophages. This gating strategy refers to Figure 6D and Figure S3F-G *B*, Gating strategy to identify lung lymphoid populations in acute and chronic infected mice. This gating strategy refers to Figure 3B-C, 6D, S3A-C and S3H. *C*, Gating strategy to identify lung myeloid populations in acute and Mtb infected mice. This gating strategy refers to Figure 3A and 5C. CD80 and CD206 gating is applied to both alveolar macrophage and interstitial macrophage populations.

**Supplementary Figures**

**A**

**B**

**C**

**Figure S1**

**K**

**J**

**L**

**M**

**E**

**H**

**I**

**G**

**D**

**F**


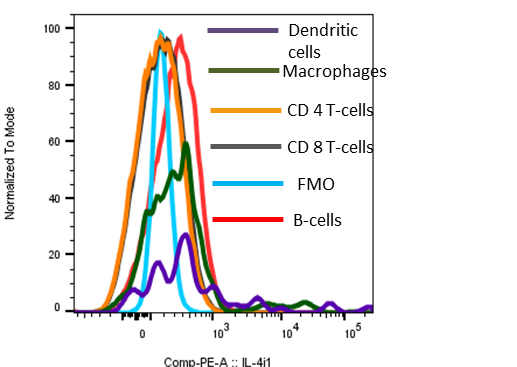


**b**

**C**

**D**


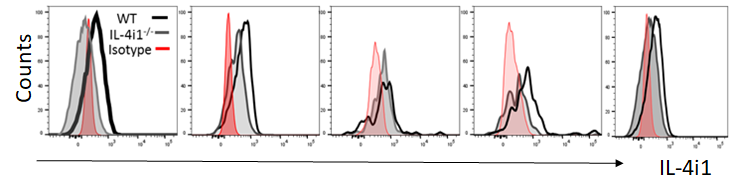


**E**

Macrophages

Dendritic cells

CD8 T-cells

CD4 T-cells

B-cells

**B**


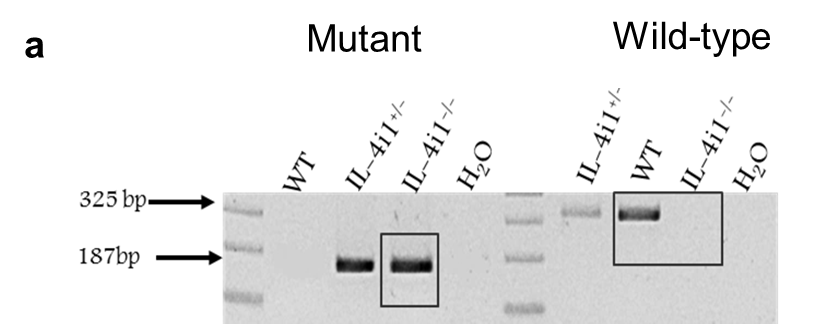


**A**

**Figure S2**

**J**

**I**

**G**

**F**

**J**

**I**

**H**

**E**

**D**

**C**

**A**

**B**

**Figure S3**


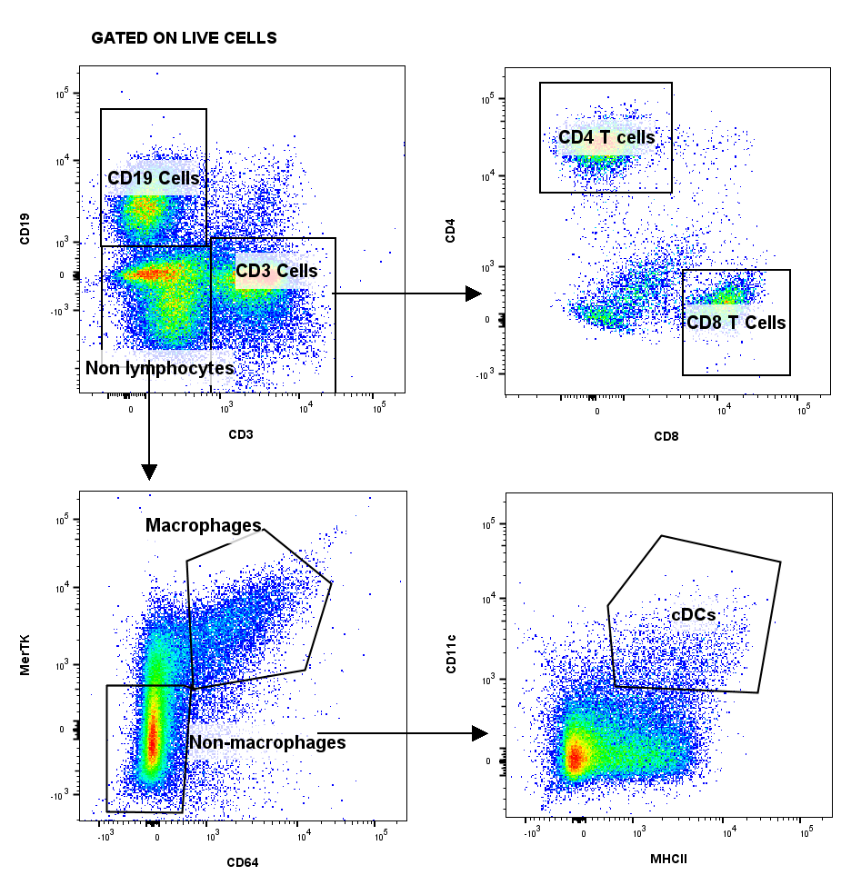


**C**

**B**

**A**

**G**

**E**

**D**

**F**

**Figure S4**


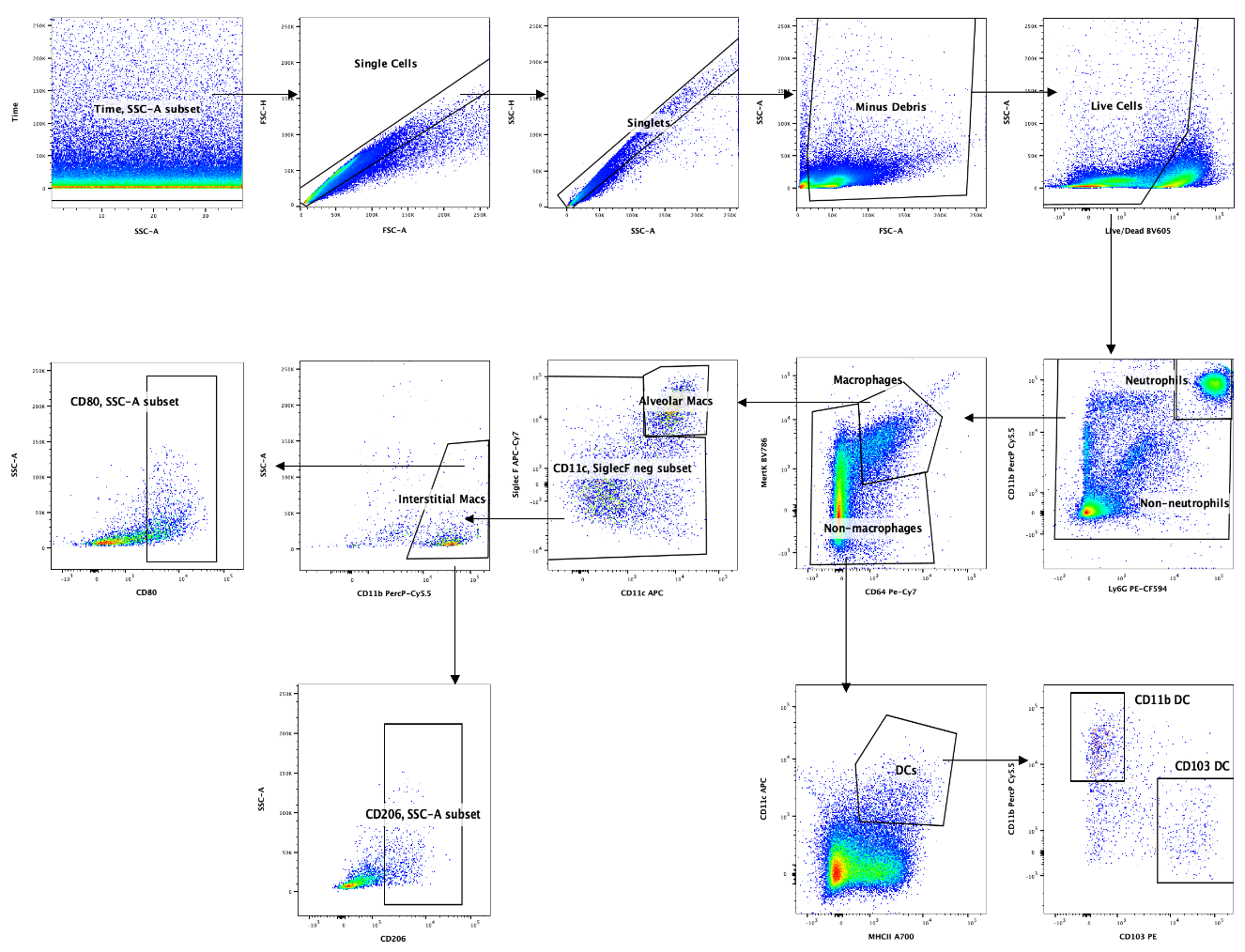


**B**

**A**


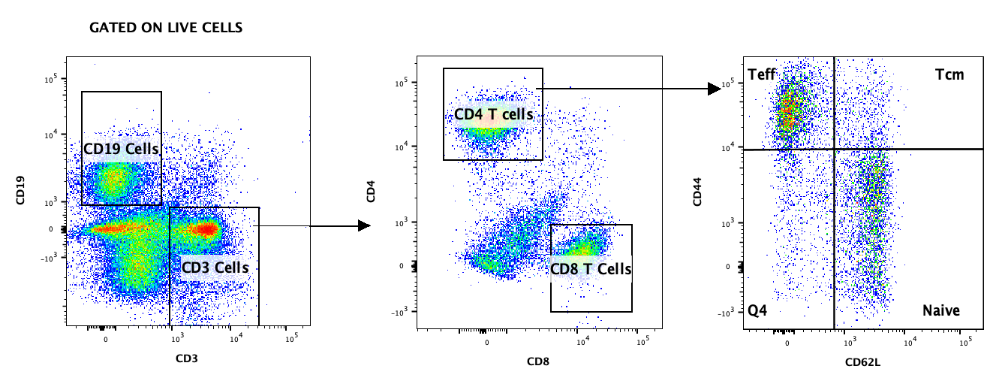

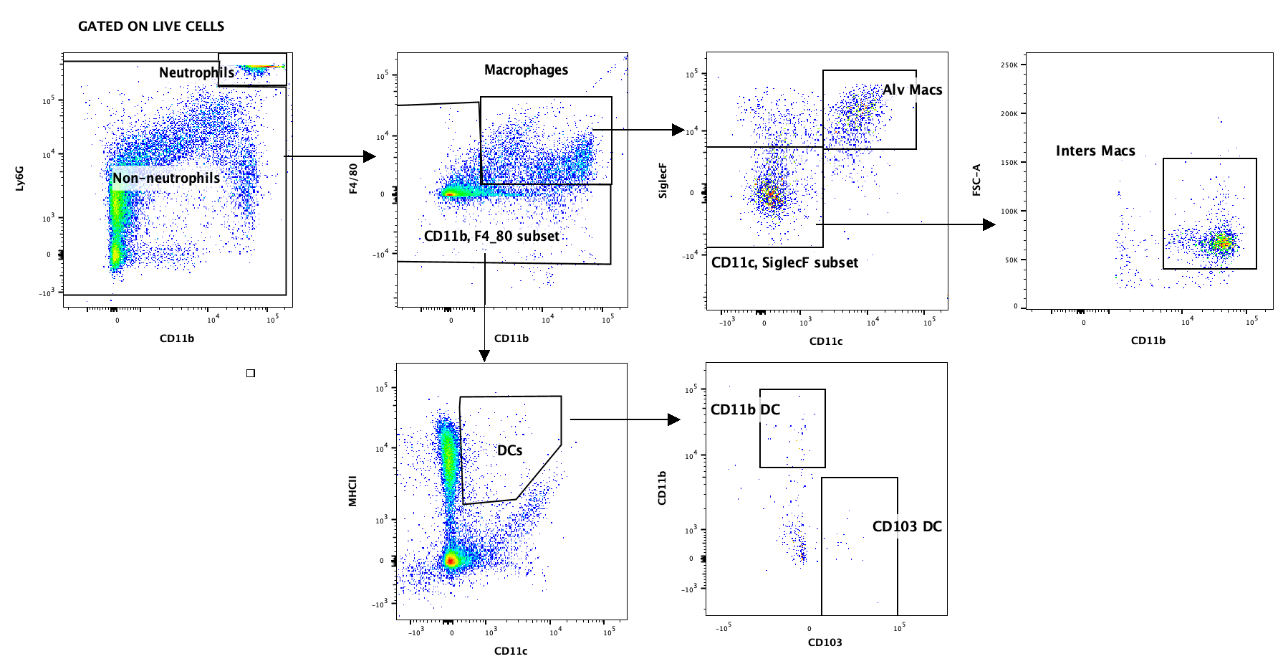


**Figure S5**

**C**

| **Gene Name** | **Forward Primer (5'-3')** | **Reverse Primer (5'-3')** |
| --- | --- | --- |
| **Il4i1** | ATTCCCCAGAGGACATCTACCA | CTGTACCGGAGTCTATCGCTCA |
| **Nos2** | AACTGCAAGAGAACGGAGAACG | AACATTCTGTGCTGTCCCAGT |
| **Ido1** | GCCTCCTATTCTGTCTTATGCAG | ATACAGTGGGGATTGCTTTGATT |
| **Ido2** | CCAGAAGGACCGTTGGAAATC | ACTGTCACTAGGATGAAGCCC |
| **Hprt** | GTTGGATATGCCCTTGAC | AGGACTAGAACACCTGCT |
| **Ifng** | GCTCTGAGACAATGAACGCT | AAAGAGATAATCTGGCTCTGC |
| **Il1b** | TGCCACCTTTTGACAGTGAT | ATGTGCTGCTGCGAGATTTG |
| **Tnf** | TCTCATCAGTTCTATGGCCC | GGGAGTAGACAAGGTACAAC |
| **Il12b** | GGAAGCACGGCAGCAGAATAA | CTTGAGGGAGAAGTAGGAATG |
| **Il6** | GTTCTCTGGGAAATCGTGGA | TGTACTCCAGGTAGCTATGG |

**Suppl Table 1: Primers used for qPCR Assay**
